# Progress and prospects of targeted therapy and immunotherapy for urachal carcinoma

**DOI:** 10.3389/fphar.2023.1199395

**Published:** 2023-05-30

**Authors:** Yang Zheng, Heling Peng, Xu Hu, Yong Ou, Dong Wang, Han Wang, Shangqing Ren

**Affiliations:** ^1^ School of Medicine, University of Electronic Science and Technology of China, Chengdu, China; ^2^ Robotic Minimally Invasive Surgery, Sichuan Academy of Medical Sciences and Sichuan Provincial People’s Hospital, Chengdu, China; ^3^ Medical Administration Department, Sichuan Academy of Medical Sciences & Sichuan Provincial People’s Hospital, Chengdu, China; ^4^ Department of Urology, Institute of Urology, West China Hospital, Sichuan University, Chengdu, China; ^5^ Department of Gastroenterology, Sichuan Academy of Medical Sciences & Sichuan Provincial People’s Hospital, Chengdu, China

**Keywords:** urachal carcinoma, immunotherapy, targeted therapy, EGFR, biomarker

## Abstract

**Introduction:** Urachal carcinoma (UrC) is a rare and aggressive disease. Systematic chemotherapy shows limited efficacy in patients with advanced disease, while targeted therapy and immunotherapy may provide a reasonable alternative for specific populations. The molecular pattern of colorectal cancer (CRC) have recently been identified; this understanding has significantly influenced the clinical management of CRC in terms of molecular-targeted therapy. Although some genetic alterations have been associated with UrC, there is still no systematic overview of the molecular profile of this rare malignancy.

**Methods:** In this review, we comprehensively discuss the molecular profile of UrC and further identify potential targets for the personalized treatment of UrC as well as immune checkpoint inhibitors that represent underlying biomarkers. A systematic literature search was carried out by searching the PubMed, EMBASE, and Web of Science databases to identify all literature related to targeted therapy and immunotherapy in urachal carcinoma from inception to February 2023.

**Results:** A total of 28 articles were eligible, and most studies included were case report sand retrospective case series. Furthermore, 420 cases of UrC were identified to analyze the association between mutations and UrC. The most commonly mutated gene in UrC was TP53 with the prevalence of 70%, followed by KRAS mutations in 28.3%, MYC mutations in 20.3%, SMAD4 mutations in 18.2% and GNAS mutations in 18%, amongst other genes.

**Discussion:** The molecular patterns of UrC and CRC are similar yet distinct. Notably, targeted therapy, especially EGFR-targeting therapy, might provide curative efficacy for patients with UrC by applying specific molecular markers. Additional potential biomarkers for the immunotherapy of UrC are mismatch repair (MMR) status and PD-L1 expression profile. In addition, combined regimens featuring targeted agents and immune checkpoint blockers might increase antitumor activity and exert better efficacy in UrC patients with specific mutational burden.

## 1 Introduction

The urachus, derived from the embryonic allantois, is a connective canal between the fetal bladder and the umbilicus during prenatal life. Subsequently, the urachus eventually degenerates to form a fibromuscular cord known as the median umbilical ligament. Failure to undergo regression may allow abnormal proliferation of the urachus and may even lead to malignances. Urachal carcinoma (UrC) is considered as a rare but aggressive malignancy that accounts for < 1% of all bladder cancers (Bruins et al., 2012) UrC is usually asymptomatic in the early stage and approximately half of patients require systematic chemotherapy to prolong their survival (Szarvas et al., 2016). Nevertheless, only a limited number of patients with advanced disease experience a response to traditional chemotherapy, and there is still no adequately powered study to confirm these benefits (Loizzo et al., 2022).

In other types of cancer, including colorectal cancer (CRC), targeted therapy has shown significant efficacy for patients with the specific expression of molecular markers (Joo et al., 2013). These encouraging outcomes have drawn significant interest from researchers with regards to the precise therapy of UrC. In recent years, some clinical series have investigated genomic alterations in UrC patients and gained promising findings in targeted therapy. Thus, in this review, we comprehensively discuss the molecular profile of UrC and further identify potential targets for the personalized treatment of UrC, In addition, given the clinical possibility of immune checkpoint inhibitors, we also discuss several biomarkers of immunotherapy.

## 2 Evidence acquisition

### 2.1 Search strategy

A systematic literature search was carried out in by searching the PubMed, EMBASE, and Web of Science databases to identify all literature related to targeted therapy and immunotherapy in UrC from inception to February 2023. The search strings mainly include the following terms: targeted therapy, immunotherapy, biomarkers, genomic alterations, urachal cancer, urachal carcinoma, and urachal adenocarcinoma. These terms were linked by using various combinations of the Boolean operators and OR. Furthermore, we performed author and citation searches as required.

### 2.2 Inclusion criteria

Studies needed to be published in English. We included gene mutations associated with UrC. Both original articles and conference abstracts were included, as were studies related to the use of targeted agents in UrC.

### 2.3 Exclusion criteria

We excluded studies that did not feature clinical data. Studies involving chemotherapy or the surgical treatment of UrC were also excluded, as were review articles and meta-analyses.

### 2.4 Selection of relevant literature

**FIGURE 1:**
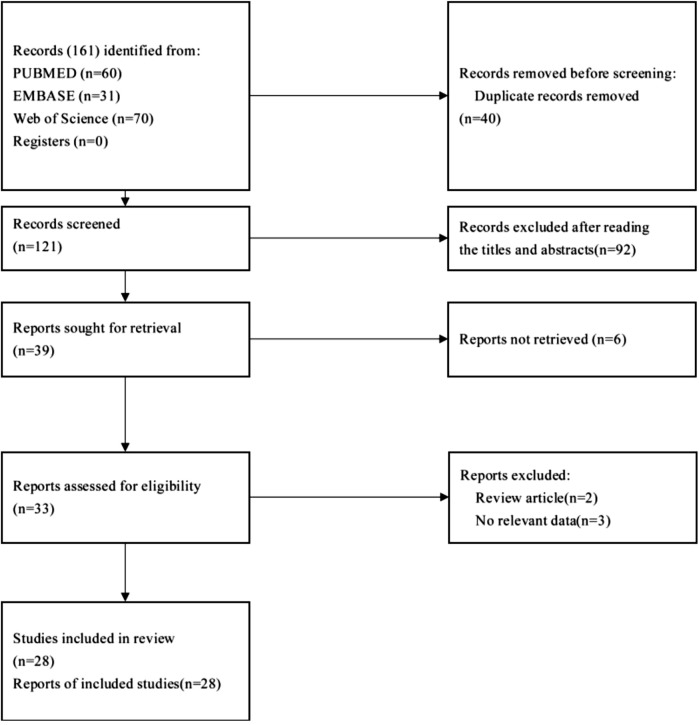
Flowchart showing how literature was selected for analysis.

The identification of eligible studies was performed based on the following two steps: initial selection by screening the title and abstract, and final inclusion after review of the full text. The detailed process used for article selection is given in Figure 1.

## 3 Evidence synthesis

### 3.1 Overall results

In this review, a total of 28 articles were eligible. Most of the included studies were case reports and retrospective case series. The response to EGFR inhibitors, PARP inhibitors, MEK inhibitors and immune checkpoint inhibitors has already been described in eight previous reports (Goss et al., 2005; Testa et al., 2014; Loh et al., 2016a; Collazo-Lorduy et al., 2016; Kardos et al., 2017; Seto et al., 2019; Yonemori et al., 2021) (listed in Table 1).

**TABLE 1:** Summary of the targeted therapy or immunotherapy received by metastatic UrC patients.

Study	Agents	Drug type	Genomic alterations	Outcome	Adverse events
Collazo-Lorduy et al. (2016)	Cetuximab	An anti-EGFR monoclonal antibody	EGFR amplification and wild-type KRAS	A 25% decrease in tumor burden for more than eight months	Acneiform rash
Goss et al. (2005)	Iressa	A selected tyrosine kinase inhibitor	EGFR amplification	A 55% decrease in tumor size for less than four weeks	NA
Loh et al. (2016a)	Trametinib	A MEK inhibitor	KRAS and GNAS mutation	NA	Pulmonary hypertension
Loh et al. (2016a)	Trametinib	A MEK inhibitor	MAP2K1 mutation	Stable disease for 10 months*	NA
Testa et al. (2014)	Sunitinib	A multi-kinase inhibitor	Unknown	Stable disease for five months	Metrorrhagia--
Seto et al. (2019)	Rucaparib	A PARP inhibitor	BRCA1 deletion	Complete response for more than 19 months	NA
Yonemori et al. (2021)	Niraparib	A PARP inhibitor	Unknown	Progressive disease*	NA
Shitara et al. (2020)	Tepotinib	A MET inhibitor	Unknown	Stable disease over 12 weeks*	NA
Kardos et al. (2017)	Atezolizumab	An anti-PD-L1 antibody	MSH6 mutation	Initial disease progression followed by regression	NA

NA: not available.

*Response assessed according to Response Evaluation Criteria in Solid Tumors (RECIST) version 1.1.

A total of 420 UrC cases were included in this study; *KRAS* was the most frequently tested gene and exhibited mutations in 28.3% (91/322) of UrC cases.

The most commonly mutated gene in UrC was *TP53* with a prevalence of 70% (170/243), followed by *KRAS* mutations in 28.3% (91/322), *MYC* mutations in 20.3% (12/59), *SMAD4* mutations in 18.2% (18/99) and *GNAS* mutations in 18% (16/89). Furthermore, other gene mutations were also found to be associated with UrC, including *APC*, *HER2*, and *BRCA* (Table 2).

**TABLE 2:** Gene mutation profile of UrC.

Author (year)	Cases, *n*	Patients with gene mutations *n* (%)																
		*KRAS*	*TP53*	*NF1*	*APC*	MSI**	*BRAF*	*EGFR*	*ERBB2*	*GNAS*	*NRAS*	*MYC*	*SMAD4*	*PIK3CA*	*PTEN*	*MET*	FGFR**	*BRCA2*
Sirintrapun et al. (2014)	7	3 (42.9)	–	–	–	3 (42.9)	0	–	–	–	–	–	–	–	–	–	–	–
Jordan et al. (2015)	10	2 (20)	–	–	0	–	–	0	2 (20)	1 (10)	–	–	–	–	–	–	–	–
Collazo-Lorduy et al. (2016)	9	2 (22.2)	7 (77.8)	0	2 (22.2)	–	0	1 (11.1)	0	1 (11.1)	1 (11.1)	–	–	1 (11.1)	–	1 (11.1)	–	–
Cornejo et al. (2016)	16	8 (50)	7 (43.8)	–	0	–	1 (6.3)	–	–	4 (25)	1 (6.3)	–	3 (18.8)	–	–	–	–	–
Lee et al. (2016)	10	2 (20)	–	–	0	–	–	0	2 (20)	1 (10)	–	–	–	–	–	–	–	–
Loh et al. (2016b)	2	1 (50)	2 (100)	–	–	–	–	–	–	1 (50)	–	–	–	–	–	–	–	–
Modos et al. (2016)	22	6 (27.3)	–	–	–	–	4 (18.2)	0	–	–	1 (4.5)	–	–	0	–	–	–	–
Singh et al. (2016)	7	2 (28.6)	4 (57.1)	3 (42.9)	3 (42.9)	–	–	–	–	–	–	–	2 (28.6)	–	–	–	–	–
Hang and Pan (2017)	12	5 (41.7)	–	–	–	0	0	–	–	0	0	–	–	–	–	–	–	–
Kardos et al. (2017)	12	–	12 (100)	3 (25)	3 (25)	7 (58.3)	–	–	–	–	–	–	–	–	–	–	–	–
Lee et al. (2017)	17	2 (11.8)	6 (35.3)	–	3 (17.6)	–	–	4 (23.5)	–	–	–	–	–	–	–	–	–	–
Modos et al. (2017)	31	8 (25.8)	–	–	–	–	5 (16.1)	0	–	–	1 (32.3)	–	–	0	–	–	–	–
Dumbrava et al. (2018)	7	–	–	–	–	–	–	–	2 (28.6)	–	–	–	–	–	–	–	–	–
Pires-Luís et al. (2018)	8	–	6 (75)	–	–	–	–	–	–	–	–	–	–	–	3 (37.5)	–	–	–
Reis et al. (2018)	70	14 (21)	46 (66)	–	–	1 (1.8)	2 (4)		–	–	1 (1)	–	–	2 (4)		1 (1)	1 (1)	–
Nagy et al. (2019)	26	8 (30.8)	19 (73.1)	–	–	3 (12)	–	–	–	–	–	6 (23.1)	–	–	–	–	3 (12)	–
Riva et al. (2019)	7	3 (42.9)	–	–	2 (28.6)	–	–	–	–	–	1 (14.3)	–	–	1 (14.3)	–	–	–	–
Maurer et al. (2020)	13	5 (38.5)	10 (76.9)	–	3 (23.1)	2 (15.4)	0	–	–	–	0	–	3 (23.1)	3 (23.1)	2 (15.4)	–	–	–
Nagy et al. (2020)	34	–	–	–	5 (15)	–	–	–	–	–	–	–	–	–	2 (6)	–	–	–
Zaleski et al. (2022)	30	9 (30)	25 (83.3)	2 (66.7)	–	–	–	–	–	8 (26.7)	–	–	7 (23.3)	3 (10)	–	–	–	–
Zhang et al. (2022)	37	–	–	–	–	3 (8.1)	–	–	–	–	–	–	–	–	–	–	–	–
Varadi et al. (2023)	33	11 (33.3)	26 (78.8)	2 (6.1)	-	2 (6.1)	–	3 (9.1)	3 (9.1)	–	–	6 (18.2)	3 (9.1)	2 (6.1)	3 (9.1)	4 (12.2)	5 (15.2)	4 (12.2)
Sum	420	91 (28.3)	170 (70)	10 (11)	21 (15.6)	21 (10)	12 (6.7)	8 (6.1)	9 (13)	16 (18)	6 (3.3)	12 (20.3)	18 (18.2)	12 (5.6)	10 (11.4)	6 (5.4)	9 (7)	4 (12.1)

*Including *MLH1*, *PMS2*, *MSH2* and MSH6. **Including *FGFR1*, *FGFR2* and *FGFR4*.-: not reported.

#### 3.1.1 Meta-analytic synthetic occurrence of gene alterations

**FIGURE 2:**
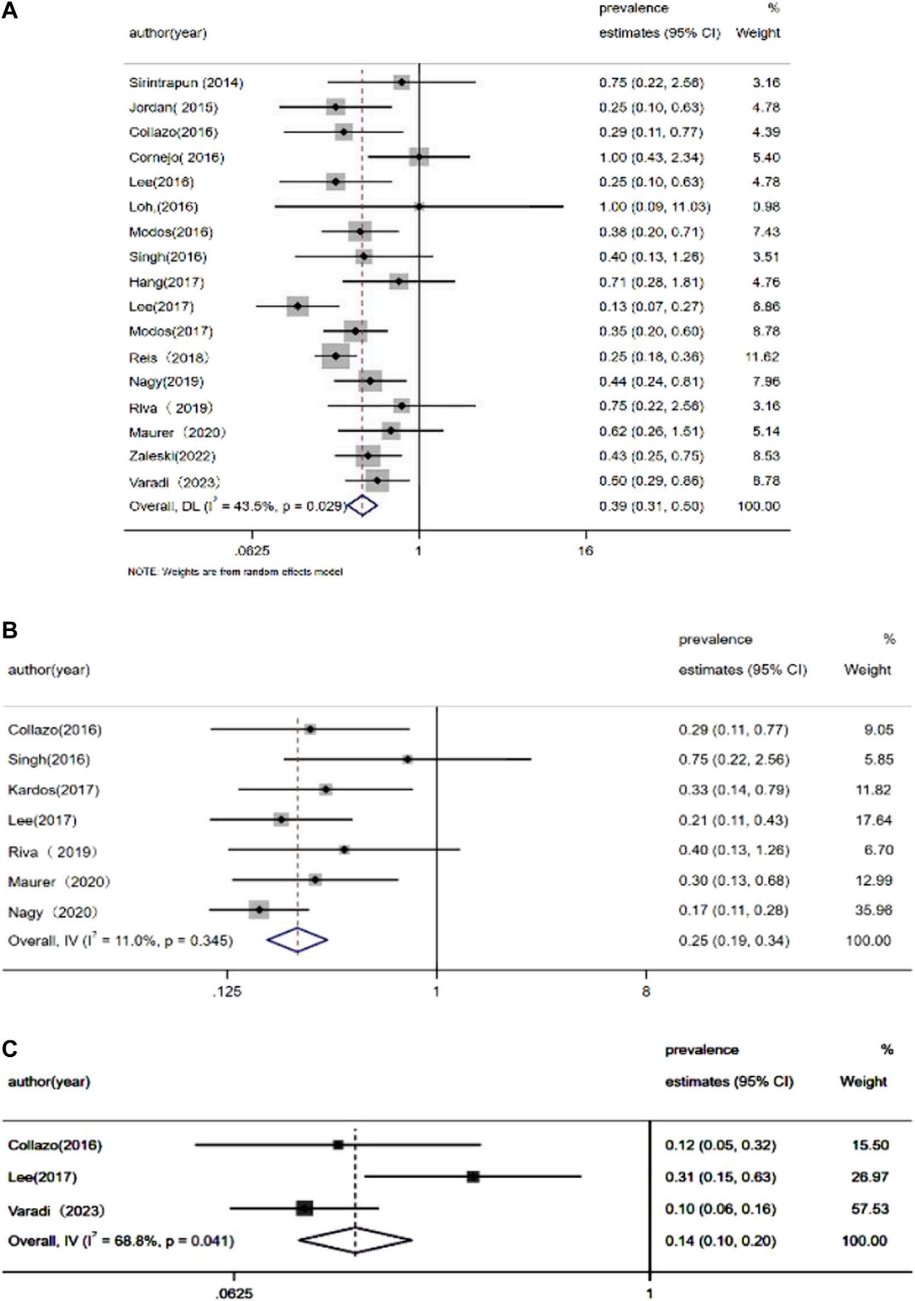
Meta-analysis of the prevalence of **(A)** *KRAS* mutations, **(B)** *APC* mutations, and **(C)** *EGFR* mutations.

The pooled prevalence estimates for several specific gene mutations are presented in Figure 2. Both Begg’s test and Egger’s test were used to analyze the publication bias of articles included in this meta-analysis. These tests demonstrated that there was no publication bias. In addition, sensitivity analyses were conducted to evaluate the robustness of the results; this analysis found that no single study affected our results (Supplementary Figure S1).

### 3.2 Targeted therapy

In the era of precision-medicine and given the limited efficacy of chemotherapy for UrC, there is an urgent need to develop molecular targeted therapy for this aggressive form of malignancy. In recent years, studies have identified recurrent gene mutations in UrC, including *EGFR*, *HER2*, and *BRCA* gene mutations, and various target agents against these aberrations have achieved promising efficacy in many types of cancers. Thus, patients with UrC who harbor specific alterations in genes such as *EGFR*, *HER2* and *BRCA* might benefit from targeted therapy.

#### 3.2.1 EGFR targeted therapy

##### 3.2.1.1 *EGFR* gene mutations

EGFR (epidermal growth factor receptor), a member of the ERBB/HER protein family, features intracellular, transmembrane, and signal-transduction domains with tyrosine kinase. The activated EGFR protein stimulates a series of downstream pathways, including the RAS/RAF/MAPK pathway and the PI3K/AKT/mTOR pathway which mainly promote cell proliferation and restrict apoptosis. However, functional *EGFR* mutations or overexpression of the EGFR protein will result in excessive cell proliferation which will eventually induce malignant transformation.

Lee et al. (2017) reported EGFR amplification in 23.5% of patients (4/17) by detecting somatic copy number aberrations. More recently, Varadi et al. (2023) described *EGFR* mutations in 9.1% (3/33) of UrC cases by applying next-generation sequencing. These authors detected two samples with amplification and one sample with a missense mutation. Both missense mutations and amplification can result in a functional EGFR protein. However, other studies failed to identify *EGFR* mutations in patients with UrC (Jordan et al., 2015; Lee et al., 2016; Modos et al., 2016; Modos et al., 2017). Statistical analysis has shown that the estimated occurrence of *EGFR* mutations was 14.1%–38.4% in patients with non-small cell lung cancer (Zhang et al., 2016). In contrast, *EGFR* aberrations only presented in 6.1% (8/132) of UrC cases while the meta-analytic pooled occurrence of *EGFR* mutations was 14% (95% CI: 10%–20%).

Thus far, two distinct therapeutic strategies have been developed to resist the pathogenic effects of mutation-activated EGFR signaling, including tyrosine kinase inhibitors (TKIs) and monoclonal antibodies. Furthermore, numerous EGFR targeted agents have been developed and approved for various types of cancers (Abourehab et al., 2021). Although no anti-EGFR antibodies and TKIs have been approved for the treatment of advanced UrC, some studies have attempted to explore the potential benefits of this novel treatment for UrC. In a recent study, one patient with metastatic disease who harbored *EGFR* amplification and wild-type *KRAS* exhibited a partial response for more than 8 months after receiving treatment with cetuximab, an anti-EGFR monoclonal antibody (Collazo-Lorduy et al., 2016). A previous phase I study evaluated the efficacy of the EGFR-TK inhibitor Iressa for various advanced tumors, including one UrC patient featuring the overexpression of EGFR; this case showed a significant reduction in tumor size by 55% following Iressa treatment (Goss et al., 2005). In addition, a patient with recurrent UrC achieved stable disease and improvement of symptoms for 5 months after receiving sunitinib, a tyrosine kinase inhibitor (Testa et al., 2014).

These promising data demonstrate that anti-EGFR-TKI therapy might also be effective for UrC. However, numerous mutations of these downstream pathways, including *KRAS*, *BRAF*, *PIK3CA*, *PTEN*, and *MET* gene mutations could increase resistance against anti-EGFR treatment (Dietel et al., 2015). Therefore, we hypothesized that the identification of actionable gene alterations in these downstream pathways is warranted before adopting EGFR targeted treatment for UrC (Wang et al., 2010; Dietel et al., 2015).

##### 3.2.1.2 Mutations of the *RAS* gene

RAS proteins, a small family of GTPase binding GTP/GDP proteins, regulate normal growth, proliferation, and differentiation in normal cells. KRAS (Kirsten rat sarcoma viral oncogene homolog), NRAS (neuroblastoma rat sarcoma viral oncogene homolog) and HRAS (Harvey rat sarcoma viral oncogene homolog) proteins are all encoded by the *RAS* gene family. When RAS aberrations occur, the RAS signaling pathway remains continuously activated without upstream stimulation of the EGFR receptor, thus resulting in abnormal cell growth, proliferation, and differentiation; this eventually results in malignant transformation. *KRAS* mutation is the most frequently altered form of the three *RAS* genes, accounting for 86% of RAS mutant cancer cases. Lee et al. (2016) reported *KRAS* mutations in 2 out of 17 patients based on whole exome sequencing and application of the Onco Scan platform while Cornejo et al. (2016) used the next-generation sequencing approach and identified *KRAS* mutations in 50% (8/16) of UrC samples; one case featured a mutation of the *NRAS* gene. More recently, Reis et al. (2018) performed targeted next-generation sequencing of 70 urachal adenocarcinomas and showed that 14 out of 70 samples (21%) had *KRAS* missense mutations. In addition, Nagy et al. (2020) reported *KRAS* mutations in 8 out of 26 (35%) samples by using the ionTorrent technology.

In the present analyses of UrC patients, we identified the presence of *KRAS* mutations in 28.3% of cases; in a previous study, *KRAS* missense mutations were found in 40% of CRC cases (Shitara et al., 2020); in addition, the meta-analytic pooled occurrence of *KRAS* mutations was 39% (95% confidence interval [CI]: 35%–50%). Sirintrapun et al. (2014) reported *KRAS* mutations in codon 12 (p.G12D, p. G12C, p. G12S, and pG12V) in some cases of UrC while Modos et al. (2016) reported that 6 out of 22 (27%) cases presented with *KRAS* alterations; 3 out of 6 *KRAS* mutations occurred in codons 61 and 146. In addition, Collazo et al. (Collazo-Lorduy et al., 2016) used a targeted exome sequencing approach and reported *KRAS* mutation at G13D and G12v in 9 UrC samples. Singh et al. (2016) also identified a G13D *KRAS* mutation in one patient with UrC. In another study, Cha et al. (Dienstmann et al., 2020) performed whole exome sequencing in adolescent and young adults with metastatic cancers; one UrC patient was identified to possess a G13D *KRAS* mutation. Using Sanger sequencing, Hang et al. (Hang and Pan, 2017) identified *KRAS* mutations in 5 out of 12 cases (p.G12V, 2 cases:p.G13D, 2 cases; p.G12R, 1 case).

Notably, in CRC, the most common *KRAS* mutation is a somatic missense mutation in codon 12 which leads to a single amino acid substitution (Zhu et al., 2021). In this review, we identified a missense mutation in codon 12 (Sirintrapun et al., 2014; Loh et al., 2016b; Collazo-Lorduy et al., 2016; Modos et al., 2016; Hang and Pan, 2017; Riva et al., 2019; Varadi et al., 2023) and codon 13 (Collazo-Lorduy et al., 2016; Singh et al., 2016; Hang and Pan, 2017; Dienstmann et al., 2020) in 23% (23/100) and 5% of UrC samples, while *KRAS* missense mutations in codon 61 (Modos et al., 2016; Riva et al., 2019) and codon 146 (Modos et al., 2016) were present in 2% and 2% of cases, respectively. Collectively, these data revealed that the most common mutation of the *KRAS* gene was the missense mutation in codon 12; this is similar to the *KRAS* mutational pattern in CRC (Cha et al., 2016).

Activated *RAS* mutations interrupt upstream transduction signals; this might result in a poor response to anti-EGFR therapy or double HER2 blockade in patients with *EGFR* or *ERBB2* amplifications (Cancer Genome Atlas Network, 2012; Siravegna et al., 2019). Given the similarity in *KRAS* mutational patterns between CRC and UrC, it is possible that the *KRAS* mutational status might also restrict targeted treatment options for this rare tumor.

Although no previous study has reported the application of KRAS inhibitors for patients with UrC, several agents targeting mutant RAS protein are available and deserve our attention. Sotorasib and adagrasib, specific and irreversible KRAS (G12C) protein inhibitors, have shown modest anti-tumor effects and tolerable toxicity in the treatment of advanced solid tumors with KRAS (G12C) mutation, including colorectal cancer (Dienstmann and Tabernero, 2016), lung cancer (Fakih et al., 2022), and pancreatic cancer (Sabari et al., 2022). A combination of these two agents with other targeted agents is under evaluation to increase potential efficacy and reduce potential drug resistance (NCT05074810, NCT04185883, and NCT03785249). In addition, several clinical trials of BI-1701963, a non-specific inhibitor of KRAS for KRAS-mutant tumors are ongoing; these trials might provide new insights for the treatment of UrC patients with KRAS mutation.

##### 3.2.1.3 Mutations of the *BRAF* gene

The *BRAF* proto-oncogene, a member of the *RAF* gene family, acts as a significant mediator in the RAS/RAF/MAPK pathway in which the BRAF protein mediates signal transduction between RAS and MAPK kinase (MAPKK/MEK1/2). Alterations in the *BRAF* gene result in the continuous activation of downstream pathways without upstream signals and therefore promotes cell proliferation and malignant transformation.

Statistical analysis has demonstrated that 30%–60% of melanoma cases harbor *BRAF* mutations; however, in our present study, only 6.7% (12/180) of UrC patients possessed *BRAF* mutations. In accordance with *RAS* mutation, many studies have shown that patients possessing *BRAF* mutations also exhibit drug resistance to cetuximab or panitumumab (anti-EGFR monoclonal antibodies) in CRC, thus suggesting the potential role of these drugs in anti-EGFR therapy for patients with UrC. Although *BRAF*-targeted agents such as vemurafenib, dabrafenib, and encorafenib, have been approved for the management of melanoma, the efficacy of BRAF inhibitors for UrC remains unclear. In our study, only four studies reported *MAP2K1* mutations in of 8.6% of 58 UrC cases, Notably, single-agent therapy involving trametinib, a *MEK* inhibitor, resulted in stable disease for 10 months in one patient with metastatic UrC and *MAP2K1* mutation (Loh et al., 2016a). This encouraging finding demonstrated that patients bearing mutations in the *MAPK* pathway particularly *MAP2K1* mutations) might acquire potential efficacy from *MEK* inhibitors. More clinical investigations are needed to validate the efficacy of *MEK* inhibitor for specific UrC patients.

##### 3.2.1.4 Mutations of the *PIK3CA gene*

The phosphoinositide 3-kinase (PI3K) pathway is one of the downstream pathways of the ERBB/HER protein family and is activated by various oncogenes and growth factor receptors [such as EGFR and human EGFR 2 (HER2)]. Furthermore, aberrantly activated PI3K signaling activity is considered as one of the characteristics of tumorigenesis. PI3CA encodes p11oalpha, a catalytic subunit of PI3K that regulates the PI3K/AKT/mTOR pathway and promotes cell survival and proliferation. PIK3CA abnormalities could result in the abnormal activation of the PI3K pathway.

In the present analyses, we found that the prevalence of *PIK3CA* mutations in UrC was 5.6% (12/215). Notably, activating *PIK3CA* alterations was related to a negative response to anti-EGFR-TKI therapy in CRC (Day et al., 2013; Strickler et al., 2023), thus suggesting that the status of *PIK3CA* genotype may be an important factor when selecting the choice of anti-EGFR therapy for patients with UrC.

Generally, the *PI3K/AKT/mTOR* pathway has two major targets: PI3K and mTOR. At present, several *PI3K* inhibitors such as idelalisib, duvelisib, and copanlisib, have been successfully marketed for the treatment of *PIK3CA*-mutant head and neck tumors, breast cancer or lymphoma. *mTOR* inhibitors, such as everolimus, are mainly used to treat advanced renal cell carcinoma. Indeed, there are no previous publications relating to the treatment of UrC with *mTOR* inhibitors or *PI3K* inhibitors.

#### 3.2.2 PARP-targeted therapy

##### 3.2.2.1 Mutations of the *BRCA* gene

Breast cancer susceptibility gene 1/2 (BRCA1/2), a tumor suppressor gene, participates in the homologous recombination repair process, and may help to safeguard genomic integrity via precise DNA repair. Furthermore, the inactivation of *BRCA1/2* genes has been observed in many forms of cancer, particularly in breast and ovarian cancer. Due to the synthetic lethality of poly (ADP-ribose) polymerase (PARP) inhibition in the presence of *BRCA1/2* mutations, tumor cells that possess BRCA1 and BRCA2-deficiency are exquisitely sensitive to treatment with PARP inhibitors (Cathomas, 2014). Furthermore, PARP inhibitors have recently gained FDA approval for patients with metastatic breast cancer and germline *BRCA* mutation (Bryant et al., 2005).

In this review, we found that only one study detected BRCA abnormalities in UrC patients with a prevalence of 12.1% (4/33). Notably, some institutions have investigated the efficacy of agents targeted to the *BRCA* gene. Seto et al. (2019) reported the successful use of rucaparib, a PARP inhibitor, in a patient with metastatic UrC who possessed a germline *BRCA1* mutation, thus suggesting that patients with *BRCA* mutations might be candidates for the use of PARP inhibitors. In a phase I dose escalation study, a UrC patient receiving niraparib was reported to experience disease progression during therapy; however, the *BRCA* status of patients with UrC was not clarified (Yonemori et al., 2021). Collectively, these data indicated that PARP inhibitors could represent a therapeutic option for UrC patients possessing BRCA deficiency.

#### 3.2.3 Mutations of the *TP53, PTEN* and *MET* genes

TP53, a widely studied tumor suppressor gene, encodes P53 protein in humans, which serves as an inhibitor of oncogenes in tumors and normal cells. Abnormal inactivation of the *TP53* gene could result in abnormal cell proliferation and could therefore lead to the development of tumors. *TP53* mutations can be found in more than half of all human cancers (Robson et al., 2017). In the present analyses, we found that *TP53* was the most frequently altered gene in UrC with a prevalence of 70% (170/243). As with other malignancies, the most common *p53* mutation in UrC patients is the missense mutation, accounting for 69.8% (60/86) of all p53 mutations. Recently, researchers have designed numerous strategies targeting *p53* mutations and developed corresponding targeting agents. In light of the *p53* status, *p53*-targeted treatment mainly include protecting normal wild-type *p53* functionality [e.g., mouse double minute 2 (MDM2) inhibitors], reactivating the wild-type functionality of mutant *p53* (e.g., mutant p53 reactivators), and eradicating mutant *p53* [e.g., histone deacetylase inhibitors, heat shock protein 90 (Hsp90) inhibitors] (Olivier et al., 2010). Nevertheless, most of these drugs are still being tested in early phase clinical trials, and no *p53*-targeted drugs have been successfully marketed thus far (Hu et al., 2021); furthermore, no previous study has reported the application of *p53*-targeted therapy for patients with UrC.

Phosphatase and tensin homolog (*PTEN*), a tumor-suppressor gene, acts as a negative mediator in the PI3K signaling pathway and can dephosphorylate PIP3 or PIP2 and prohibit the activation of PI3K and therefore interrupt this pathway. In our study, *PTEN* mutations were identified in 11.4% (10/88) of UrC cases. As with *PIK3CA* mutations, abnormal *PTEN* alterations were shown to result in a negative response to anti-*EGFR*-TKI therapy in CRC (Hassin and Oren, 2023). Notably, Nagy et al. (2020) reported the presence of *PTEN* mutations in 9.1% (3/33) of 33 UrC samples, whereas the loss of PTEN protein was detected in 20% (6/30) of UrC cases, as determined by immunohistochemistry. The higher rate of PTEN loss at the protein level showed that epigenetic mechanisms are engaged in the downregulation of PTEN. These results suggested that the immunohistochemical analysis of PTEN protein might have significant potential to predict drug resistance to anti-EGFR therapy.

The MET tyrosine kinase receptor, also known as the hepatocyte growth factor receptor, is dependent on the *MET* proto-oncogene. MET can act as a driver of malignant progression but also as a mediator of drug resistance in various cancers. Varadi et al. (2023) used a next-generation sequencing approach and identified *MET* mutations in 12% (4/3) of UrC cases. In addition, these authors detected two samples with functional mutations; one of these was a dysfunctional mutation while the status of the other mutation remained unknown.

In a phase I trial of tepotinib, a *MET* inhibitor, one patient with metastatic UrC receiving tepotinib treatment achieved stable disease over 12 weeks with a progression-free survival (PFS) of 12.9 months; however, the *MET* status of this patient was unknown (Frattini et al., 2007). The overall prevalence of *MET* mutations in UrC was previously reported to be 5.4% (6/112); this is similar to that of *MET* mutations in CRC (2%–4%) (Zhang et al., 2018). Furthermore, studies have shown that *MET* amplification can resist the efficacy of anti-*EGFR* therapy in CRC (Luraghi et al., 2014), thus suggesting *MET* status might also represents a predictor of efficacy for anti-*EGFR* therapy in UrC.

#### 3.2.4 Mutations of *APC*

Adenomatous polyposis coli (APC) plays a negative role in the β-catenin/Wnt signaling pathway that regulates cell proliferation and differentiation. APC interacts with and degrades ß-catenin, a transcriptional regulator. An inactivating *APC* mutation results in the release of pathway inhibition and leads to the abnormal accumulation of nuclear β-catenin, eventually facilitating mitosis and cell proliferation. Nagy et al. (2020) used a targeted next-generation sequencing approach and identified APC mutations in 14.7% (5/34) of UrC cases; these authors detected two cases with truncating mutations. In another study, Singh et al. (2016) described APC mutations in 43% (3/7) of UrC cases, including one case with a nonsense mutation, one case with a frameshift mutation, and one case with a deletion. Both the deletion and frameshift mutations resulted in the inactivation of APC protein. In another study, Collazo et al. (Collazo-Lorduy et al., 2016) reported truncating mutations in the *APC* gene (R1450*, R554*) in 22% (2/9) of UrC cases while Lee et al. (2017) described the presence of a frameshift deletion and a stop-gain single nucleotide variant (E1093*).

After summarizing all available data relating to APC status, an estimated 15.6% (21/135) of UrC cases possessed *APC* mutations; this is very different to the rate of *APC* mutation observed in cases of CRC (80%) (Cha et al., 2016), thus demonstrating that the Wnt pathway might play a role in the pathogenesis of UrC in only a relatively small proportion of cases.

### 3.3 Immunotherapy

Another proficient anti-cancer strategy is targeting tumoral immune-escape mechanisms, in which inhibitors of the programmed cell death-1 (PD-1)/programmed cell death-ligand 1 (PD-L1) checkpoint and cytotoxic T lymphocyte-associated protein-4 (CTLA-4) are of particular therapeutic interest. PD-1/PD-L1 inhibitors have proven efficacious in several types of advanced cancer, including melanoma, non-small-cell lung cancer, renal cell carcinoma, and bladder cancer (Yu, 2018). Ipilimumaba, a CTLA-4 inhibitor, has been approved for the treatment of patients with metastatic melanoma in 2011 (Hodi et al., 2010)**.** As immune checkpoint inhibitors have been recently approved for treatment of advanced bladder cancers (Eckstein et al., 2019), the use of immunotherapy for UrC cases might represent a new treatment option for this rare form of malignancy.

#### 3.3.1 Biomarkers of immunotherapy

As with other solid tumors, specific biomarkers are urgently needed to identify patients with UrC who might respond to immunotherapy. The presence of MMR deficiency and positive PD-L1 expression on tumor cells might be potential biomarkers of efficacy for the use of PD-1/PD-L1 blockades in patients with UrC. However, no previous study has investigated biomarkers for anti-CTLA therapy in UrC.

##### 3.3.1.1 PD-L1 expression status

The expression of the PD-L1 on tumor cells has validated and significant efficacy for PD-1 inhibition in cases of non-small-cell lung cancer (Mok et al., 2019). However, the predictive value of PD-L1 expression for the treatment of UrC with PD1 inhibitors is currently unknown, although some institutions have shared their experience with regards to PD-L1 expression profile. For example, Reis et al. (2018) performed PD-L1 analyses using clone 22C3; in 10 out of 63 cases (15.9%), a specific level of PD-L1 expression was detected in tumor cells. Positive PD-L1 expression was associated with a shorter PFS (*p* = 0.002). In another study, Maurer et al. (2020) evaluated PD-LI expression in glandular bladder tumors and stained all available samples (including samples from three UrC patients) with four different anti-PD-L1 antibody clones (28–8, SP142, SP263, and 22C3); none of the three UrC patients tested were positive for PD-L1 expressing immune cells and tumor cells.

In another study, Zhang et al. (2022) used immunohistochemistry to investigate the characteristics of the immune microenvironment in 37 UrC patients. These authors found that the proportion of immune cells that were positive for PD-L1 protein expression was 35.14% (13/37). Only one case (2.78%) was found to possess tumor cells that were positive for PD-L1 membranous expression. In addition, immune cells that were positive for PD-L1 expression were only marginally associated with a short overall survival (OS) (*p* = 0.3700) and disease free survival (DFS) (*p* = 0.5400) in patients with UrC. Notably, no significant correlation was identified between the protein expression of PD-L1 in immune cells, histological type, and tumor stage, After summarizing all available data from 103 UrC patients with PD-L1 expression levels, 11 (10.7%) were positive for PD-L1 expression. However, 35.14% (13/37) of cases expressed PD-L1 on tumor-associated immune cells,

##### 3.3.1.2 Mismatch repair deficiency status

Mismatch repair pathways serve as pivotal regulators in identifying and repairing mismatched nucleotides during DNA replication or genetic recombination and therefore guarantees genomic integrity and stability. The MMR system consists of a series of DNA mismatch repair proteins, including MLH1, PMS2, MSH2, and MSH6; these can be identified by immunohistochemical approaches in clinical practice. A deficiency of MMR (dMMR) proteins may lead to spontaneous hypermutation alterations and therefore result in microsatellite instability (MSI) (Zhao et al., 2019). Furthermore, research has revealed high consistency between dMMR and MSI (Cicek et al., 2011). Furthermore, dMMR status has been reported in approximately 15% of CRC cases (Zhao et al., 2019) whereas the loss of MMR protein was detected in 10% of 210 UrC samples. In another study, Sirintrapun et al. (2014) described one case involving the loss of MSH2, MSH6 and PMS2 proteins. In another study, Kardos et al. (2017) used a targeted exon sequencing method to identify the presence of dMMR in 25% (3/12) of urachal tumors, including two cases with MSH6 loss and one case with the loss of MSH2 and polymerase epsilon complex (POLE). More recently, Zhang et al. (2022) found that 8.1% (3/37) of UrC patients suffered from dMMR, as determined by IHC staining. However, Hang et al. (Hang and Pan, 2017) also used immunohistochemistry and demonstrated that MMR proteins (MLH1, MSH2, MSH6 and PMS2) were preserved in all UrC samples tested. Similarly, Reis et al. (2018) investigated 56 cases by performing MSI analyses and identified only one case that was negative for MSH2 expression. Notably, patients with MMR defects exhibited a considerable response to PD-1 blockade regardless of tumor type (Dudley et al., 2016) Pembrolizumab, an immune checkpoint inhibitor, was approved in 2017 for the treatment of MMR defects in solid tumors (Marcus et al., 2019). Similarly, Kardos et al. (2017) reported one UrC patient harboring a MSH6 mutation who received atezolizumab, an anti-PD-L1 antibody. This patient benefited from the treatment, thus suggesting that dMMR status may represent a favorable biomarker for immunotherapy in UrC.

## 4 Discussion

Given that urachal cancer and colorectal cancer share the same embryological region of origin (Upadhyay and Kukkady, 2003), many studies have compared the genomic profile of UrC with CRC in an attempt to identify potential therapeutic targets for these rare tumors. After summarizing all available data, we observed both similarity and disparity between CRC and UrC at the molecular level (Supplementary Table S1). The high mutational prevalence of *TP53* (70%), *KRAS* (28.3%), *MYC* (20.3%), and *SMAD4* (18.2%), suggests that the cell cycle pathway, RAS signaling pathway, and TGF-β pathway, all participate in the pathogenesis of UrC in a large proportion of cases. This finding is comparable to those with CRC; APC mutations are considered as the distinct molecular signatures of CRC as they occur in over 80% of cases; only 15.6% of UrC cases possess APC alterations, thus indicating that the Wnt pathway might be less significant in the malignant transformation of UrC. In addition, both in CRC and UrC, a missense mutation in codon 12 was the most frequently altered mutation in the *KRAS* gene. Both CRC and UrC are associated with a low occurrence of *EGFR*, *BRCA*, *PTEN*, *MET* and *BRAF* gene mutations. However, the incidence of *HER 2* mutations in UrC (13%) is significantly higher than that in CRC (3%–5%).

Notably, some studies have explored the association between mutational status and prognosis. For example, Nagy et al. (2020) reported that neither APC mutations nor positive nuclear ß-catenin were correlated with overall survival. In the same cohort, the presence of *PTEN* mutations and the loss of PTEN protein did not exert a significant effect on overall survival. In addition, although patients with *KRAS* mutations are usually related to a poor prognosis in CRC or lung adenocarcinoma (Uras et al., 2020), the prognostic role of *KRAS* mutations in patients with UrC still remains controversial. Sirintrapun et al. (2014), conducted *KRAS* mutation testing in a series of seven patients with advanced-stage UrC and revealed that patients possessing *KRAS* mutations had a better overall survival (mean survival: 101.7 *versus* 6.5 months; *p* = 0.035). However, Zaleski et al. (2022) used next-generation sequencing to analyze the genomic alterations of 30 cases and found that 30% of cases (9/30) possessed *KRAS* mutations; furthermore, the presence of *KRAS* mutations predicted a worse overall survival (*p* = 0.006). Given that this particular study only involved a low number of cases with genomic alterations, these results should be interpreted cautiously; their prognostic value for UrC needs to be investigated further.

As UrC shares some genomic alterations with CRC, therapeutic strategies that target CRC might represent a useful reference for the development of strategies for UrC. Currently, several agents have been developed to target the EGFR, including Cetuximab and panitumumab; these demonstrate significant efficacy for patients with metastatic CRC. However, some patients with specific mutations, such as *KRAS*, *NRAS*, *PTEN*, *PIK3CA*, and *MET* are known to respond poorly to anti-EGFR therapy. Notably, most of the poorly responding patients involve problems with the downstream pathways of EGFR, including the RAS/RAF/MAPK pathway, and the PI3K/AKT/mTOR pathway In the present analyses, a high proportion of UrC cases possessed functional gene alterations which played a pivotal role in the EGFR signaling pathway. In addition, some studies have attempted to investigate the potential benefit of EGFR-TKI treatment for UrC and reported encouraging benefits, thus indicating that anti-EGFR therapy might be an effective treatment option for patients with chemotherapy-refractory UrC. Therefore, the genotype of the affected genes may need to be considered before selecting anti-EGFR therapy, even if there is no evidence to validate their role in anti-EGFR therapy. In addition, since a moderate proportion of UrC patients possessed dMMR (12.1%) or *MAP2K1* mutations (8.6%), PARP-targeted therapy and MEK-targeted therapy might also represent curative options for patients harboring dMMR or *MAP2K1* alterations, although there is no convincing evidence to validate their efficacy at present.

However, several deficiencies of targeted therapy of urachal carcinoma should be recognized. Firstly, drug-related toxicity is a significant factor that can determine whether targeted therapy could be adopted. Goss et al. (2005) conducted a phase I study evaluating the safety, toxicity and other clinical parameters of Iressa treatment for several advanced solid tumors with EGFR overexpression and noted that the dose-limiting toxicity of Iressa mainly included skin rash and diarrhea, both of which were reversible after dose reduction or treatment interruption; furthermore, no significant hematological toxicity was detected. In another study (Collazo-Lorduy et al., 2016), an acneiform rash was observed in a UrC patient treated with cetuximab; this was managed with focal medication without treatment discontinuation. Furthermore, in a case series of metastatic UrC by Loh et al. (2016a), one case discontinued trametinib treatment owing to severe adverse events, while another case interrupted sorafenib management due to a diffuse skin rash (grade 3 adverse event). These data suggested that treatment-related toxicity could limit the efficacy of targeted agents and therefore restrain the application of targeted therapy in UrC patients, at least to some extent. Second, given drug resistance was observed in other malignancies following targeted therapy such as anti-EGFR therapy, potential drug resistance might also be a limitation of targeted therapy in UrC. On this context, a combination of multi-targeted-therapy regimens might make a difference.

With regards to immunotherapy, although both mismatch repair deficiency status and positive PD-L1 expression are considered to represent favorable biomarkers for PD-1/PD-L1 inhibitors, little is known about their role in UrC. In addition, no clinical studies have confirmed the efficacy of immunotherapy for the treatment of UrC. This might be due to the rarity of this aggressive disease; thus, it is difficult to conduct clinical trials. Notably, previous studies have demonstrated that TKI treatment could regulate tumor-related immune response through the tumor microenvironment (Madeddu et al., 2022). Furthermore, preclinical studies enrolling patients with EGFR mutant non-small cell lung cancer have suggested that *EGFR* mutations could contribute to cancer immune escape through the PD-1/PD-L1 pathway (Bruno and Dowlati, 2019). In addition, several specific mutated genes including *BRAF* and *PI3KCA* are often accompanied by dMMR in CRC (Van Cutsem et al., 2019). Based on these findings, it can be hypothesized that a combined regimen of targeted agents and immune checkpoint blockers might increase antitumor activity and exert better efficacy in UrC patients with specific mutational burden. Currently, in a phase I study enrolling patients with metastatic UrC and rare GU malignancies, combination therapies have shown acceptable toxicities and encouraging results (Nadal et al., 2017) (ClinicalTrials.gov Identifier: NCT02496208). In addition, an ongoing phase II clinical trial is attempting to validate the efficacy of a multi-targeted-therapeutic regimen involving the combination of nivolumab (a PD-1 inhibitor), ipilimumab (an anti-CTLA-4 inhibitor) and cabozantinib (a non-specific MET inhibitor) in patients with genitourinary tumors including urachal adenocarcinoma (ClinicalTrials.gov Identifier: NCT03866382).

There are several limitations to this study that need to be considered. First, due to the low incidence of UrC, our current knowledge about this disease is mainly derived from case reports and retrospective case series; this is not suitable for the acquisition of high-level evidence for the clinical management of patients with UrC. Indeed, for most rare tumors, numerous pragmatic challenges are inevitable, including the lack of a comprehensive understanding of disease pathogenesis and the inadequate interest and funding available for drug development. Furthermore, due to the rarity of UrC, it is difficult to recruit a sufficient number of patients to conduct clinical trials, let alone establish a standard system to test new interventions. However, some alternative methods may be able to address these problems (Billingham et al., 2016). For example, maximizing the study duration for clinical studies related to time-to-event outcome could improve statistical power in a small sample size; and using Bayesian framework in a randomized controlled study could significantly reduce uncertainty relating to treatment effect size for the limited patient population. Second, due to heterogeneity in the gene sequencing panels and techniques adopted by different studies, some functional gene alterations may not have been detected. Therefore, we should consider the full range and prevalence of different genomic mutations in UrC. Thus, a standardized gene sequencing technique with a comprehensive gene panel is mandatory for UrC as this will help to accurately define a comprehensive picture of genetic alterations and their frequency, thus providing clinical reference for future clinical investigations. In addition, we performed an additional meta-synthesis of the prevalence of gene mutations; however, owing to the lack of detailed patient characteristics, it was not possible to perform subgroup or survival analysis. Finally, as most of the included studies were case series or case reports, it was not possible to perform quality assessments.

## 5 Conclusion

Our analyses found that the molecular patterns of UrC and CRC are distinct yet similar. Notably, targeted therapy, especially EGFR-targeting therapy, might provide curative efficacy for patients with UrC who are positive for specific molecular markers. Furthermore, target sequencing of relevant functional genes needs to be considered before selecting anti-EGFR therapy for UrC. In addition, MMR status and PD-L1 expression profile appear to represent potential biomarkers for immunotherapy in UrC.

## Figures and Tables

**Table:** 

**UrC**	urachal carcinoma
**CRC**	colorectal cancer
**TP53**	tumor-suppressor gene tumor protein p53
**KRAS**	Kirsten rat sarcoma viral oncogene homolog
**MYC**	myelocytomatosis oncogene
**SMAD4**	mothers against decapentaplegic homolog 4
**GNAS**	G protein alpha(s)
**EGFR**	epidermal growth factor receptor
**TKI**	tyrosine kinase inhibitors
**APC**	adenomatous polyposis coli
**HER2**	human epidermal growth factor receptor 2
**BRCA**	breast cancer susceptibility gene
**PARP**	poly (ADP-ribose) polymerase
**RAS**	rat sarcoma viral oncogene homolog
**BRAF**	v-raf murine sarcoma viral oncogene homolog B
**MAPK**	mitogen-activated protein kinases
**PI3K**	phosphoinositide 3-kinase
**AKT**	V-akt murine thymoma viral oncogene homolog
**mTOR**	mechanistic target of rapamycin
**MMR**	mismatch repair
**PD-1**	programmed cell death-1
**PD-L1**	programmed cell death-Ligand 1
**PIK3CA**	phosphatidylinositol-4, 5-bisphosphate 3-kinase catalytic subunit alpha
**PTEN**	phosphatase and tensin homolog
**MET**	METproto-oncogene receptor tyrosine kinase
**NF1**	neurofibromatosis type 1
**MSI**	microsatellite instability
**NRAS**	neuroblastoma rat sarcoma viral oncogene homolog
**FGFR**	fibroblast growth factor receptor
**HRA**	Harvey rat sarcoma viral oncogene homolog
**dMMR**	deficiency of MMR
**MLH1**	mismatch repair gene mutL homolog 1
**PMS2**	postmeiotic segregation increased 2
**MSH2**	mutS homolog 2
**MSH6**	mutS homolog 6
**TGF-β**	transforming growth factor-beta
**CTLA-4**	cytotoxic T lymphocyte-associated protein 4
**POLE**	polymerase epsilon complex
**MDM2**	mouse double minute 2
**Hsp90**	Heat shock protein 90

## Data Availability

The raw data supporting the conclusion of this article will be made available by the authors, without undue reservation.
