# Supplementary material for: Progress and prospects of targeted therapy and immunotherapy for urachal carcinoma
Source: Front Pharmacol. 2023 May 30;14:1199395. doi: 10.3389/fphar.2023.1199395 (PMC10267743; doi:10.3389/fphar.2023.1199395)
Supplement: Supplementary file 1 [file Table1.docx]

Supplemental table 1 Mutation profile of UrC and CRC

|  | mutation profile | | | | | | | | | | | |
| --- | --- | --- | --- | --- | --- | --- | --- | --- | --- | --- | --- | --- |
|  | *Tp53* | *KRAS* | *SMAD4* | *MSI* | *APC* | *ERBB2* | *PTEN* | *BRAF* | *MET* | *EGFR* | *PIK3CA* | *NRAS* |
| UrC | 70.0% | 28.3% | 18.2% | 10.0% | 15.6% | 13.0% | 11.4% | 6.7% | 5.4% | 6.1% | 5.6% | 3.3% |
| CRC | 50.0%(1) | 40.0%(2) | 30.0%(3) | 15.0%(4) | >80.0%(5) | 3-5.0%(6) | 4-10.0%(5) | 10%-15.0%(7) | 2-4.0%(8) | 1.0%(7) | <12.0%(9) | 4.0%(2) |

1 Li XL, Zhou J, Chen ZR and Chng WJ (2015). P53 mutations in colorectal cancer - molecular pathogenesis and pharmacological reactivation. World journal of gastroenterology; 21(1): 84-93.doi:10.3748/wjg.v21.i1.84

2 Dienstmann R, Connor K and Byrne AT (2020). Precision Therapy in RAS Mutant Colorectal Cancer. Gastroenterology; 158(4): 806-11.doi:10.1053/j.gastro.2019.12.051

3 Wang H, Stephens B, Von Hoff DD and Han H (2009). Identification and characterization of a novel anticancer agent with selectivity against deleted in pancreatic cancer locus 4 (DPC4)-deficient pancreatic and colon cancer cells. Pancreas; 38(5): 551-7.doi:10.1097/MPA.0b013e31819d7415

4 Zhao P, Li L, Jiang X and Li Q (2019). Mismatch repair deficiency/microsatellite instability-high as a predictor for anti-PD-1/PD-L1 immunotherapy efficacy. Journal of hematology & oncology; 12(1): 54.doi:10.1186/s13045-019-0738-1

5 (2012). Comprehensive molecular characterization of human colon and rectal cancer. Nature; 487(7407): 330-7.doi:10.1038/nature11252

6 La Salvia A, Lopez-Gomez V and Garcia-Carbonero R (2019). HER2-targeted therapy: an emerging strategy in advanced colorectal cancer. Expert opinion on investigational drugs; 28(1): 29-38.doi:10.1080/13543784.2019.1555583

7 Li J, Ma X, Chakravarti D, Shalapour S and DePinho RA (2021). Genetic and biological hallmarks of colorectal cancer. Genes & development; 35(11-12): 787-820.doi:10.1101/gad.348226.120

8 Zhang M, Li G, Sun X, Ni S, Tan C, Xu M, et al. (2018). MET amplification, expression, and exon 14 mutations in colorectal adenocarcinoma. Human pathology; 77: 108-15.doi:10.1016/j.humpath.2018.03.024

9 Day FL, Jorissen RN, Lipton L, Mouradov D, Sakthianandeswaren A, Christie M, et al. (2013). PIK3CA and PTEN gene and exon mutation-specific clinicopathologic and molecular associations in colorectal cancer. Clinical cancer research : an official journal of the American Association for Cancer Research; 19(12): 3285-96.doi:10.1158/1078-0432.Ccr-12-3614
