# Supplementary figures and images for: Progress and prospects of targeted therapy and immunotherapy for urachal carcinoma
Source: Front Pharmacol. 2023 May 30;14:1199395. doi: 10.3389/fphar.2023.1199395 (PMC10267743; doi:10.3389/fphar.2023.1199395)

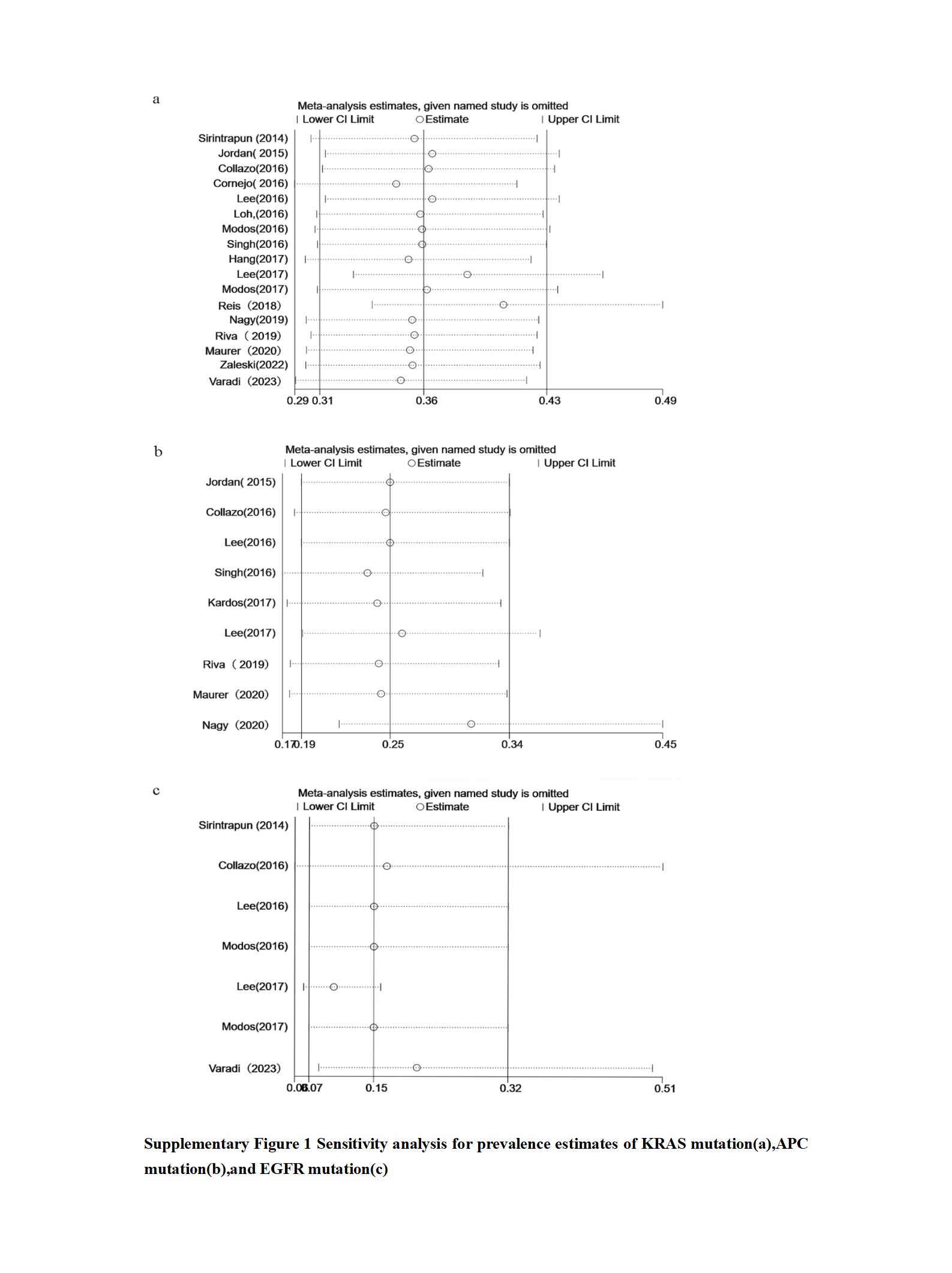

Supplement: Supplementary file 2 [file Image1.jpeg]
